# A novel HCC prognosis predictor PDSS1 affects the cell cycle through the STAT3 signaling pathway in HCC

**DOI:** 10.3389/fonc.2022.927468

**Published:** 2022-07-28

**Authors:** Zuqin Rao, Heng Li, Wenchao Yao, Qiang Wang, Biao Ma, Dongbo Xue, Xianzhi Meng

**Affiliations:** ^1^ Department of General Surgery, The First Affiliated Hospital of Harbin Medical University, Harbin, China; ^2^ Key Laboratory of Hepatosplenic Surgery, Ministry of Education, The First Affiliated Hospital of Harbin Medical University, Harbin, China; ^3^ Department of Comprehensive Surgery, The First Affiliated Hospital of University of Science and Technology of China (USTC) West District/Anhui Provincial Cancer Hospital, Hefei, China

**Keywords:** PDSS1, immune infiltration, prognosis, cell cycle, STAT3

## Abstract

Decaprenyl diphosphate synthase subunit 1 (PDSS1) is closely related to a variety of human diseases, but its expression pattern and biological function in HCC have not been studied to date.

**Methods:**

The expression level of PDSS1 was analyzed using the TCGA and GEO databases. The relationships between PDSS1 and patient clinicopathological characteristics were verified based on TCGA clinical data. Additionally, the co-expressed genes of PDSS1were investigated and Gene Set Enrichment Analysis (GSEA) was conducted using LinkedOmics. Next, the association between PDSS1 and immune infiltration was determined using version 1.34.0 of the GSVA package. EdU assay, colony-formation assay, transwell assay, wound-healing assay, and flow cytometry analysis were used to assess the effect of PDSS1 on the cell phenotype.

**Results:**

PDSS1 was upregulated in HCC compared with adjacent tissues. High PDSS1 in HCC was associated with poor overall survival, disease-specific survival, and progress-free interval. Results suggested that PDSS1 may activate multiple oncogenic pathways in HCC, especially those involved in the cell cycle. The expression of PDSS1 was significantly related to Th2 cells, TFH, T helper cells, NK CD56bright cells, cytotoxic cells, DC, CD8 T cells, and neutrophils. PDSS1 knockdown inhibited cell proliferation, cell cycle, migration and invasion. Furthermore, PDSS1 acted as an oncogene through the STAT3 signaling pathway.

**Conclusion:**

Our study reveals that a high level of PDSS1 is significantly correlated with poor patient prognosis and immune cell infiltration in HCC. PDSS1 may be a novel biomarker and potential therapeutic target for HCC.

## Introduction

Liver cancer (primary liver cancer; PLC) includes hepatocellular carcinoma (HCC), intrahepatic cholangiocarcinoma, and mixed liver cancer; about 90% of liver cancers are HCC (1). HCC is the seventh most common malignant tumor and the fourth most common cause of tumor death (2). More and more clinical data show that the incidence of liver cancer is on the rise and that patients tend to be younger. To date, the cause of HCC remains unclear. It is currently believed that HCC is the result of genetic and environmental factors. HCC is closely correlated to hepatitis B virus (HBV), long-term alcoholism, nonalcoholic fatty liver disease, hepatitis C virus (HCV) infection, eating food contaminated with aflatoxin B1, various types of liver cirrhosis, etc. (3). The occurrence of HCC is the result of a combination of individual genetic background, immune mechanism, viral factors, and environmental factors (4, 5). The onset of HCC is hidden, and there are no obvious symptoms in the early stage. In many patients, it is not found until it is already in the middle to late stages; thus, the best time for treatment is missed. Early diagnosis and treatment are critical to the prognosis of HCC. Therefore, it is crucial to find a novel molecular target to elucidate the mechanism of HCC.

Many studies have shown that there are metabolic abnormalities in tumors (6). In our study, we were interested in the decaprenyl diphosphate synthase subunit 1 (PDSS1), which catalyzes the condensation of farnesyl diphosphate and isopentenyl diphosphate to produce pentenyl diphosphates of different chain lengths and is involved in the determination of ubiquinone side chains (7). Optic atrophy and sensorineural deafness were shown to be due to missense PDSS1 mutations in coenzyme Q10 synthesis (8). Genetic variation of PDSS1 in the metabolic pathway of ketone bodies was found to be significantly related to cutaneous melanoma prognosis (9). Recently, a study revealed that PDSS1 can regulate the levels of coenzyme Q10 and calcium ions in cells, and induce phosphorylation of STAT3. The phosphorylated STAT3 enters the nucleus and activates the STAT3 signaling pathway to promote metastasis in triple-negative breast cancer (TNBC) (10). Nevertheless, the expression pattern, biological function, and potential mechanism of PDSS1 in HCC are still unknown.

STAT3 is one of the most complex regulators of transcription. Constitutive activation of STAT3 has been reported in many types of tumors and depends on mechanisms such as hyperactivation of receptors for pro-oncogenic cytokines and growth factors, loss of negative regulation, and excessive cytokine stimulation (11). In the liver, STAT3 is mainly activated by IL- 6 or IL- 22 and regulates acute phase response proteins, liver regeneration, hepatoprotection and gluconeogenesis. STAT3 has been considered as a bona fide oncogene that counteracts tumor-suppressive STAT1 activities (12, 13).

In this research, we verified the expression of PDSS1 and its correlation with patient prognosis in HCC by using many databases and websites, including the Cancer Genome Atlas (TCGA), Gene Expression Omnibus (GEO), GEPIA, and a Kaplan–Meier plotter. The LinkedOmics database and GSEA were used to identify the role of PDSS1 in HCC. The association of PDSS1 and immune infiltration was then analyzed using the GSVA package. Moreover, we silenced PDSS1 expression and detected the effect of this intervention on the proliferation, migration, and invasion of HCC cells. Finally, we examined the potential molecular mechanism of PDSS1 in promoting the progression of HCC.

## Material and methods

### PDSS1 expression analysis in cancer

The HCCDB (http://lifeome.net/database/hccdb/home.html) and Omcomine (https://oncomine.org/resource/login.html) were used to investigate PDSS1 expression in HCC (14, 15). The c-BioPortal (http://cbioportal.org) was used to investigate PDSS1 mutations and CNV. The mRNA expression data of PDSS1 were downloaded from TCGA and GEO databases. Normal sample information was obtained from the GTEx database. This study did not require ethical approval, because all data had been collected from publicly available database.

### Survival analysis

The associations between patient prognosis (OS, DSS, PFI) and PDSS1 expression were determined using GEPIA (http://gepia.cancer-pku.cn/) and the Kaplan–Meier plotter (http://kmplot.com/analysis/). The univariate and multivariate cox regression models were analyzed using R software.

### GSEA enrichment and co-expressed genes analysis

The genes co-expressed with PDSS1 in HCC were explored *via* the LinkedOmics database. These genes were used in KEGG/GO pathway analysis and GSEA enrichment analysis.

### Drug sensitivity analysis and cancer pathway activity

Using the TCGA HCC dataset, PDSS1-involved cancer pathway analysis was performed using GSCALite (http://bioinfo.life.hust.edu.cn/web/GSCALite/) (16), and PDSS1 drug sensitivity analysis was performed using Genomics of Drug Sensitivity in Cancer (GDSC) and the Cancer Therapeutics Response Portal (CTRP).

### Cell culture and transfection

Human hepatocellular cancer cell lines (HepG2, LM3, Huh7, Hep3B, and SMMC-7721) were purchased from the American Type Culture Collection (ATCC, Virginia, USA). All HCC cells were cultured in DMEM (Gibco, USA) with 10% fetal bovine serum. Cells were incubated at 37°C with 5% carbon dioxide. LM3 and Hep3B cells were transfected with siRNA (Tongyong Biotech, China) using OPTI-MEM (Invitrogen) and Lipofectamine 8000 (Beyotime Biotechnology, China) according to the manufacturer’s instructions. The targets of the PDSS1 siRNA sequence were as follows: siRNA#1: 5′-GCACGAAUUGGAAAUACAATT-3′ and 5′-UUGUAUUUCCAAUUCGUGCTT-3′; siRNA#2: 5′-GGUUCACGAUGACGUUAUUTT-3′and 5′-AAUAACGUCAUCGUGAACCTT-3′.

### Construction of shPDSS1 plasmid

Select siRNA#1 as the target sequence, reverse complement the sequence and connect with CTCGAG as loop. Then, the recognition sites of EcoR I and Age I were added before and after the sequence. Finally, insert the above sequence into the pLKO.1 cloning vector.

### Quantitative real-time PCR

The total RNA of cells was isolated using TRIzol reagent (Invitrogen, USA) according to the manufacturer’s manual. Next, cDNA was reverse transcribed from RNA using PrimeScript RT Master Mix (Takara). The qPCR was performed with a SYBR Green PCR kit (Takara). GAPDH served as the internal control. The primer sequences were as follows (5’–3’): PDSS1 forward: CGCCATAGCCTTAATTGCAGA, reverse: GAACTTGCATCGTCAATAACGTC.

GAPDH forward: TGCACCACCAACTGCTTAGC, reverse: GGCATGGACTGTGGTCATGAG. Relative expression levels were normalized to the internal control.

### Western blotting

Cell lines were lysed with RIPA buffer containing a cocktail (Beyotime Biotechnology, China). Cell proteins were separated by SDS-PAGE and transferred to PVDF membranes (Millipore, MA, USA), and then blocked for 1 h at room temperature. Membranes were incubated with primary antibodies overnight at 4°C. Membranes were washed and incubated with secondary antibody for 1 h. Protein signals were detected using an ECL detection system. The antibodies were as follows: anti-PDSS1 (AP53614, Abcepta), anti-STAT3 (ab68153, Abcam), anti-p-STAT3 (ab76315, Abcam), anti-HMGA2 (20795-1-AP, Proteintech), anti-MYC (10828-1-AP, Proteintech), anti-cyclin D1 (26939-1-AP, Proteintech), and anti-beta tubulin (10094-1-AP, Proteintech).

### EdU assay

LM3 and Hep3B cells were plated onto a 24-well plate, and then incubated with EdU diluent for 2 h at 37°C (KaiJi Biotechnology). After being fixed and permeabilized, cells were stained with EdU mixture and DAPI. Finally, results were observed *via* fluorescence microscopy.

### Colony formation assay

LM3 and Hep3B cells were seeded onto a 6-well plate with 500 cells per well and incubated for two weeks. After being fixed for 30 minutes and stained with crystal violet for 20 minutes, the number of colonies was counted.

### Cell cycle assay

LM3 and Hep3B cells were harvested 48 h after transfection with siRNA. All cells were then fixed overnight with 70% cold ethanol. Second day, cells were incubated with 500ul PI staining for 30 min. Finally, cell cycle was tested using cell flow cytometer (Beckman Coulter, Life Sciences).

### Migration and invasion assay

A transwell chamber was used to test cell migration and invasion. LM3 and Hep3B cells were seeded at 2×10^4^ into the upper chamber (Corning, NY) containing 200μL serum-free DMEM. The lower chamber was supplied with 800μL DMEM containing 10% FBS. After incubation for 24 h, the cells that migrated underneath the membrane were fixed and stained. Pictures were then taken under a microscope, and the number of migratory cells was calculated. For the invasion assay, the same cells were seeded into the upper chamber. The upper chamber was coated with Matrigel (BD, USA) and the above procedure was followed. A wound-healing assay was also used to investigate cellular migration and invasion.

### Animal experiment

Male athymic BALB/c nude mice (4–5 weeks old) were used for animal study. For xenograft tumor assay, 1 × 10^6^ transfected LM3 cells were prepared for subcutaneous injection to mice for 28 days. The animal experiments obtained permission through the Animal Ethics and Welfare Committee (AEWC) of the First Affiliated Hospital of Harbin Medical University.

### Statistical analysis

Data obtained from TCGA and GEO databases were analyzed using R software (version 3.6.1). SPSS 20.0 software and GraphPad Prism 8 were used to process the experimental results. Data are reported as means ± standard deviation. Results between different groups were compared using Student’s t-test. A difference of *p* < 0.05 was considered statistically significant.

## Results

### PDSS1 was highly expressed in HCC

**Figure 1:**
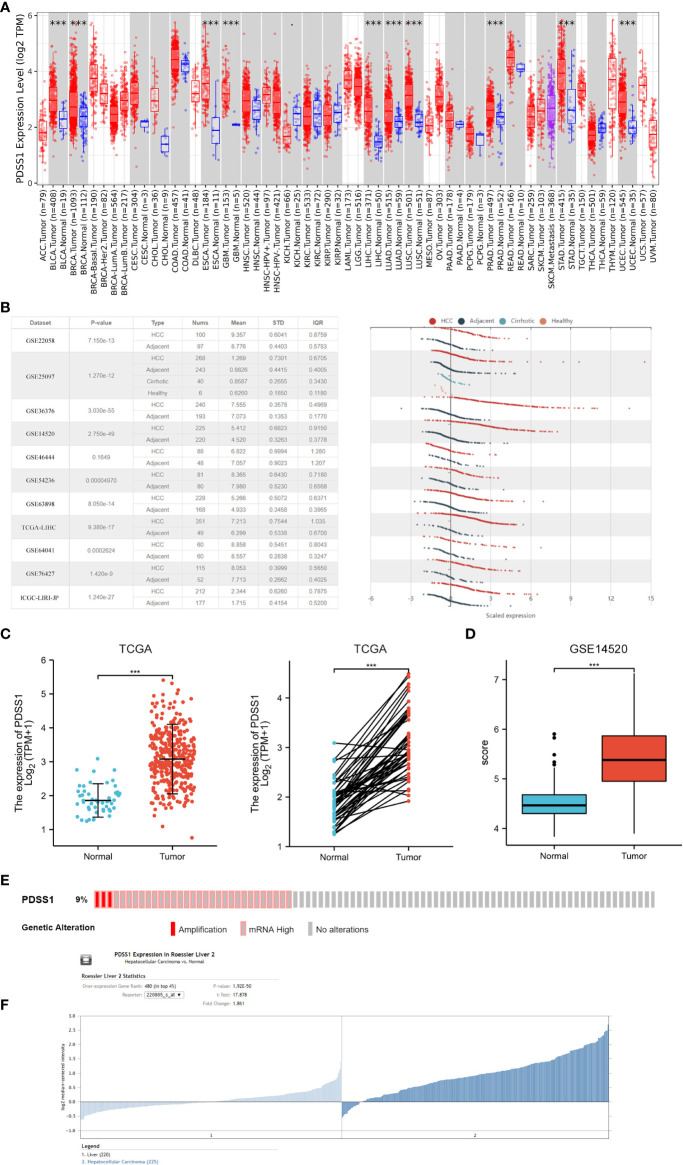
The expression of PDSS1 in different cancers. **(A)** Increased or decreased PDSS1 of different cancers compared to normal tissues in the TCGA and GTEx database. **(B)** The mRNA expression of PDSS1 in tumor tissues and adjacent tissues according to the HCCDB database. **(C)** PDSS1 expression in tumor and normal tissues from TCGA. Left for unpaired and right for paired. **(D, F)** PDSS1 expression in tumor and normal tissues in GSE14520. **(E)** The genomic alterations of PDSS1 in HCC based on c-BioPortal database. ***p < 0.001.

Based on the data from the TCGA and GTEx databases, we first evaluated PDSS1 expression in pan-cancers using GEPIA. The results showed that PDSS1 is significantly overexpressed in liver hepatocellular carcinoma (LIHC), bladder urothelial carcinoma (BLCA), esophageal carcinoma (ESCA), breast infiltrating carcinoma (BRCA), glioblastoma multiforme (GBM), uterine corpus endometrial carcinoma (UCEC), lung adenocarcinoma (LUAD), lung squamous cell carcinoma (LUSC), pancreatic cancer (PAAD), and gastric cancer (STAD) (*p*<0.05;**Figure 1A**). The HCCDB and Oncomine database are tumor databases that contain 3917 samples from the TCGA and GEO databases. We observed that the level of PDSS1 was upregulated in HCC compared to adjacent tissues based on data from the HCCDB and Oncomine databases (**Figures 1B**, **C**). GSE14520 database as a validation data set once again verified the expression pattern of PDSS1 in HCC (**Figures 1D**, **F**). To understand the mutations of PDSS1, cBioPortal was applied to detect the DNA sequence information of liver cancer patients. The results showed that PDSS1 genetic alteration occurs in 9% of patients with HCC (**Figure 1E**).

### The correlation of PDSS1 expression with prognosis and clinicopathological characteristics in HCC patients

**Figure 2:**
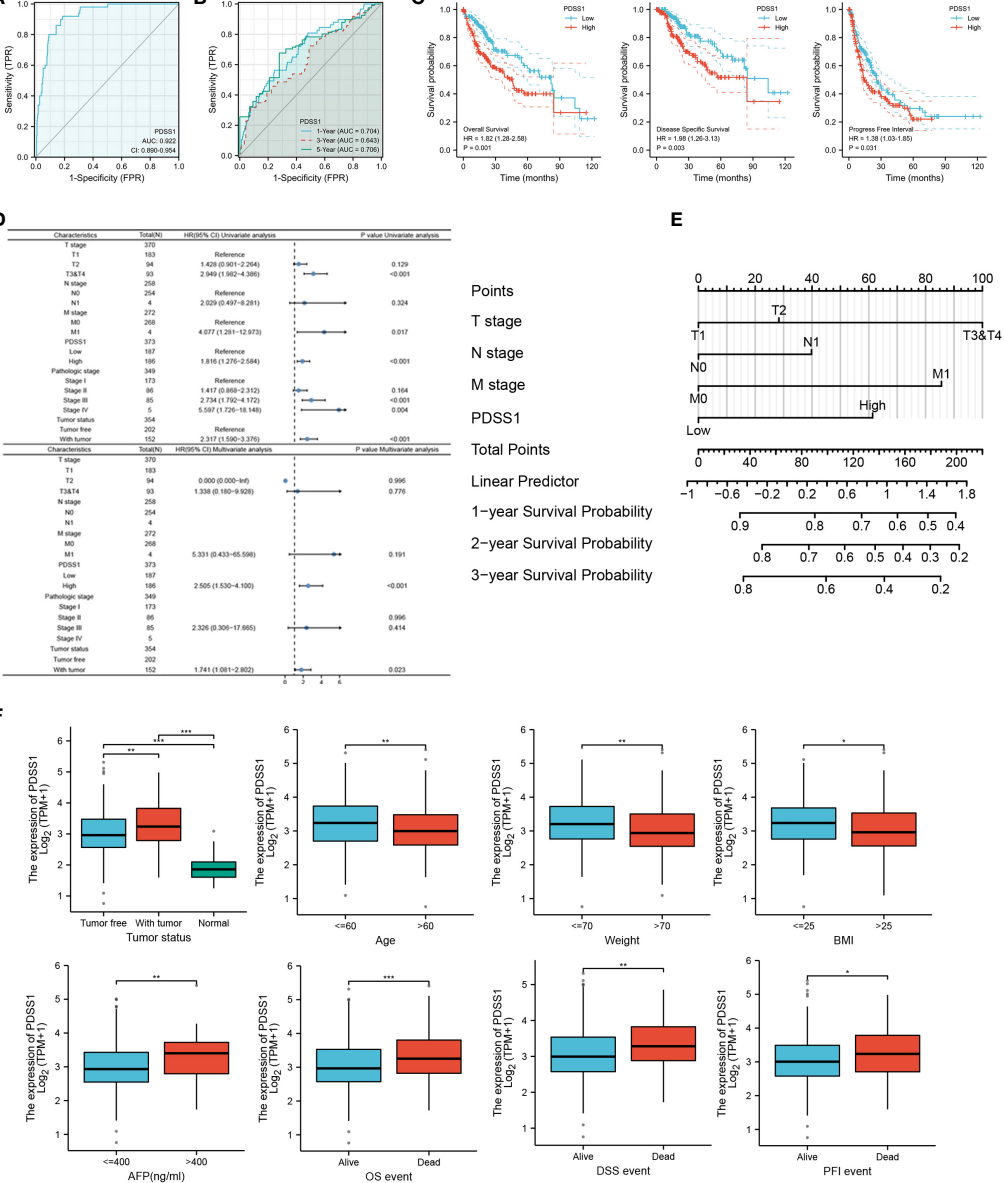
Expression of PDSS1 was correlated with prognosis of HCC patients. **(A, B)** ROC curve to test the value of PDSS1 to identify HCC. **(C)** overall survival, disease specific survival and progress free interval in HCC using GEPIA database. **(D)** Univariate and multivariate Cox regression analysis identified the association of PDSS1 with the clinical factors with OS in TCGA database. **(E)** Nomogram map showed the correlation between PDSS1 expression and prognosis. **(F)** Correlation between expression of PDSS1 and clinic-pathological features. *p < 0.05, **p < 0.01, ***p < 0.001.

To investigate the relationship of PDSS1 expression and patient prognosis, we performed receiver operating characteristic (ROC) analysis and found that the AUC values of PDSS1 were 0.922, 0.704 (1 year), 0.634 (3 years), and 0.706 (5 years). This result indicates that the upregulation of PDSS1 expression in HCC has diagnostic significance (**Figures 2A**, **B**). In addition, we used the GEPIA website to analyze the association between PDSS1 expression level and HCC patient clinical outcome. We found that HCC patients with high expression levels of PDSS1 show poor overall survival (OS) (*p* = 0.001), disease-specific survival (DSS) (*p* = 0.003), and progress-free interval (PFI) (*p* = 0.031) (**Figure 2C**). Using univariate Cox regression model analysis, we found that a high expression level of PDSS1 was significantly related to poor prognosis (HR = 1.816; 95% CI: 1.276 – 2.584; *p* < 0.001), as well T stage (HR = 2.949; 95% CI: 1.982 – 4.386; *p* < 0.001), M stage (HR = 4.077; 95% CI: 1.281 – 12.973; *p* = 0.017), pathological stage III (HR = 2.734; 95% CI: 1.792 – 4.172; *p* < 0.001), pathological stage IV (HR = 5.597; 95% CI: 1.726 – 18.148; *p* = 0.004), and tumor status (HR = 2.317; 95% CI: 1.590– 3.376; *p* < 0.001). Subsequently, these characteristics were incorporated into a multivariate Cox regression analysis; the result showed that high PDSS1 expression (HR = 2.505; 95% CI: 1.530 – 4.100; *p* < 0.001) and tumor status (HR = 1.741; 95% CI: 1.081 – 2.802; *p* = 0.023) were independent prognostic factors for poor prognosis (**Figure 2D**). Nomogram mapping also indicated that high PDSS1 was closely associated with poor prognosis in HCC patients (**Figure 2E**). Moreover, based on the TCGA database, we divided liver cancer patients into high and low groups according to their PDSS1 expression levels, and analyzed the relationship between PDSS1 and clinicopathological characteristics. The results showed that a high level of PDSS1 was positively related to race (*p* = 0.038), body mass index (BMI, *p=* 0.043), histologic grade (*p* = 0.003), AFP (*p* < 0.001), and OS event (*p* = 0.023) (**Figure 2F** and **Table S1**).

### Co-expressed genes and enrichment analysis

**Figure 3:**
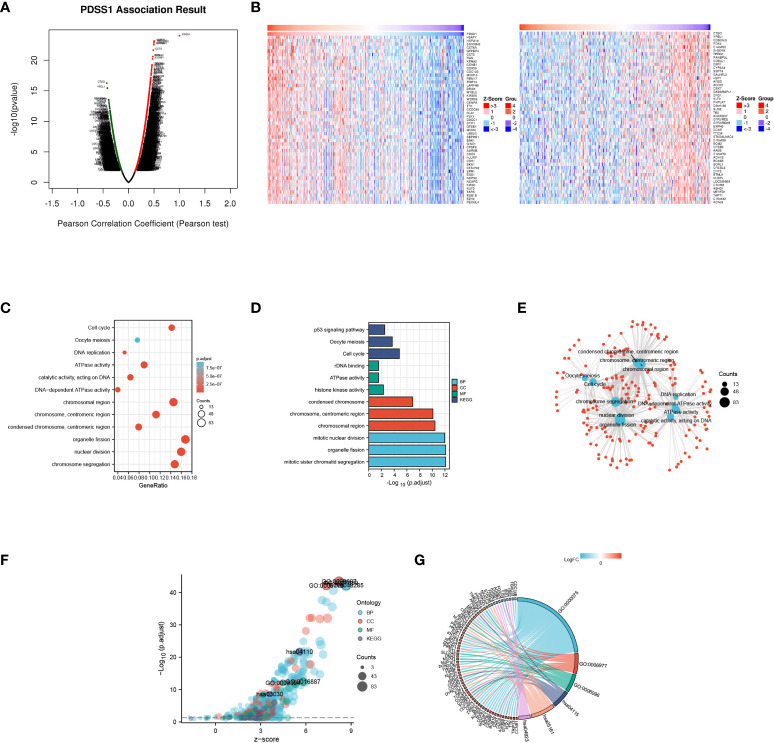
Genes co-expressed with PDSS1 in HCC. **(A)** Highly correlated genes identified by the Pearson test in the HCC cohort. **(B)** The heat maps showing top 50 genes positively and negatively correlated with PDSS1 in HCC (left: positively correlated genes; right: negatively correlated genes). **(C-F)** Significantly enriched GO annotations and KEGG pathways of the genes co-expressed with PDSS1 in HCC. **(G)** The co-expressed genes related to cell cycle checkpoint, cell cycle arrest, glycolytic process, p53 signaling pathway, hepatitis B, Regulation of lipolysis in adipocytes.

**Figure 4:**
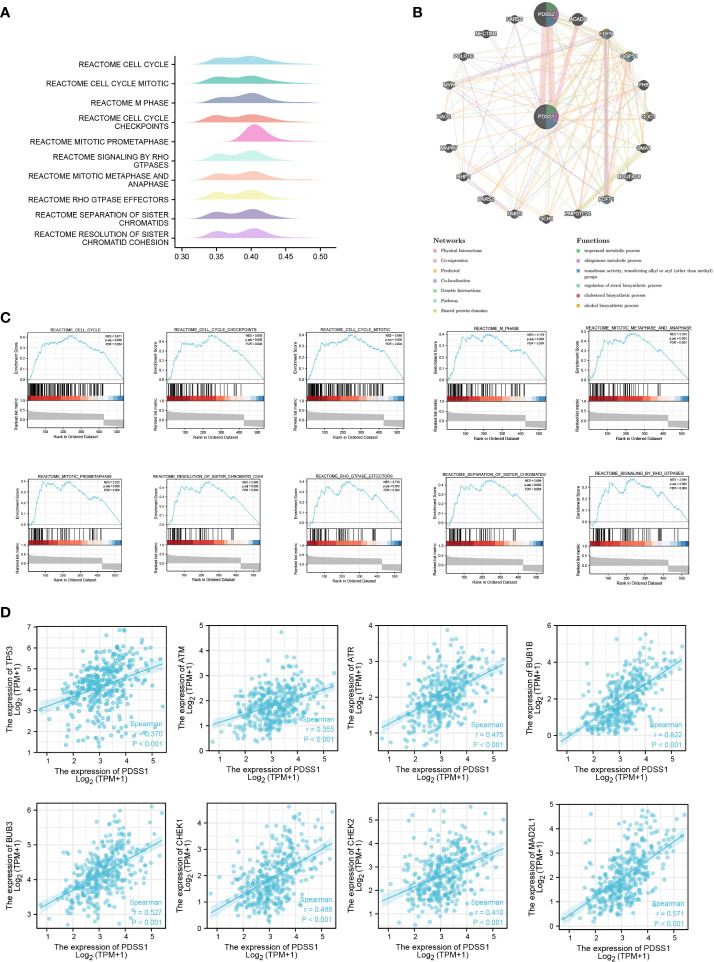
The potential PDSS1-regulated pathway. **(A, C)** Reactome analysis revealed the pathway enriched in PDSS1 high and low expression phenotype. **(B)** Protein–protein interaction (PPI) network and functional analysis indicating the gene sets enriched in the target network of PDSS1. **(D)** Correlation between expression of PDSS1 and cell cycle checkpoint related genes.

To explore the potential mechanism of the PDSS1 gene in HCC, the LinkedOmics database was used to detect co-expressed genes of PDSS1. As showed in **Figure 3A**, 487 genes (right red dots) were positively correlated with the expression of PDSS1, while 126 genes (left green dots) showed a significant negative association with PDSS1 (*p* <0.05, | r | >0.3). The heat map presents the top 50 genes that were positively and negatively correlated with PDSS1 (**Figure 3B**). Next, we explored the molecular functions and physiological pathways involving PDSS1 based on these co-expressed genes. GSEA was used for GO functional, KEGG pathway, and reactome analyses. The results indicated that PDSS1 is mainly related to DNA replication, mitotic sister chromatid segregation, p53 signaling pathway, and cell cycle (**Figures 3C**–**E**). The reactome analysis further showed that co-expressed genes of PDSS1 may be involved in the cell cycle, M phase, cell cycle checkpoints, and separation of sister chromatids (**Figures 3F**, **G**). The reactome analysis further showed that co-expressed genes of PDSS1 may be involved in the cell cycle, M phase, cell cycle checkpoints, and separation of sister chromatids (**Figures 4A**, **C**). The above results suggest that high expression of PDSS1 may activate multiple oncogenic pathways in HCC, especially the cell proliferation pathway. Therefore, we analyzed the association between PDSS1 and key genes related to cell cycle checkpoints, such as TP53, ATM, ATR, BUB1B, BUB3, CHEK1, CHEK2, and MAD2L1. The results showed that PDSS1 has a significant positive correlation with these genes (*p*<0.001, **Figure 4D**). Using a protein–protein interaction (PPI) network, we found the proteins that interact with PDSS1, as shown in **Figure 4B**. These proteins are mainly involved in the isoprenoid metabolic process, regulation of sterol biosynthetic process, ubiquinone metabolic process, cholesterol biosynthetic process, transferase activity, or alcohol biosynthetic process transferring alkyl or aryl. This indicates that PDSS1 may affect the occurrence and development of tumors by changing these biological metabolic processes and provides new insights for the study of tumor metabolism.

### Immune infiltration analysis for PDSS1

**Figure 5:**
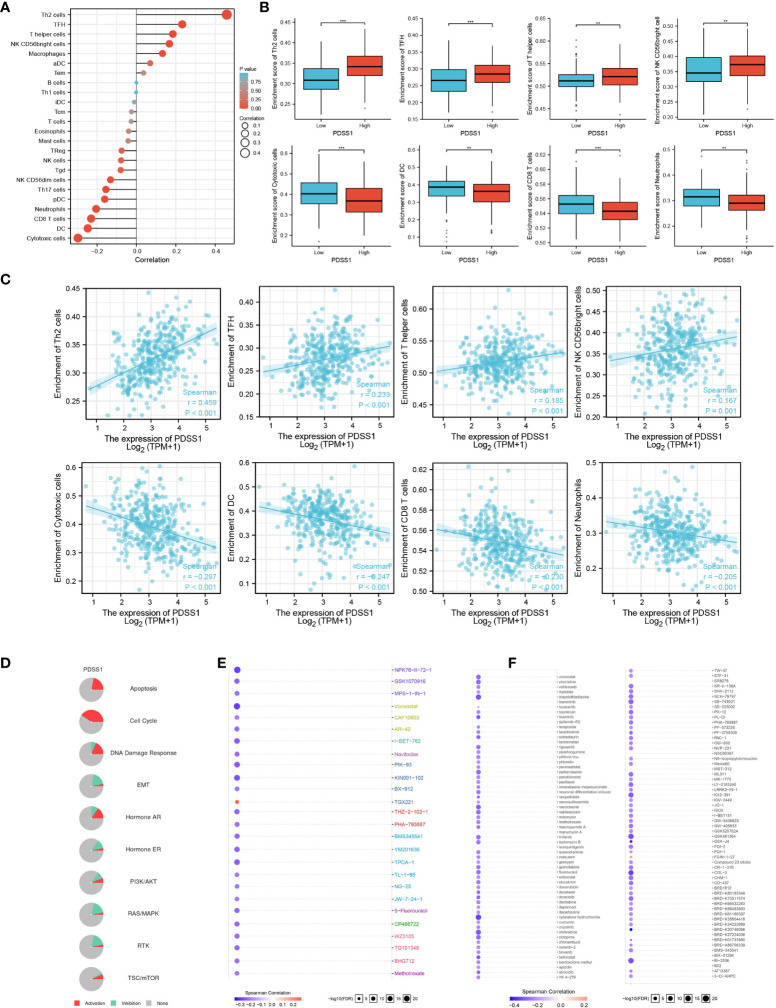
The role of PDSS1 in immune infiltration, cancer pathways and the drug sensitivity. **(A-C)** Correlation between expression of PDSS1 and immune cells infiltration. **(D)** PDSS1-related Cancer pathway activity. PDSS1-related drug sensitivity using GDSC **(E)** and CTRP **(F)** database. **p < 0.01, ***p < 0.001.

To explore the role of PDSS1 in the tumor immune microenvironment, we used immune infiltration analysis to analyze the infiltration of different immune cells following changes in the expression level of PDSS1. Immunocytes include Th2 cells, TFH, T helper cells, NK CD56bright cells, macrophages, aDC, Tem, B cells, Th1 cells, iDC, Tcm, T cells. eosinophils, mast cells, Treg, NK cells, Tgd, NK CD56dim cells, Th17 cells, pDC, neutrophils, CD8 T cells, DC, and cytotoxic cells. We found that PDSS1 was significantly positively related to Th2 cells (r = 0.459, *p*<0.001), TFH (r = 0.233, *p* < 0.001), T helper cells (r = 0.185, *p*<0.001), NK CD56bright cells (r = 0.167, *p*<0.001), and was negatively correlated to cytotoxic cells (r = - 0.297, *p*<0.001), DC (r = - 0.247, *p*<0.001), CD8 T cells (r = - 0.230, *p*<0.001), neutrophils (r = - 0.205, *p*<0.001). This showed that PDSS1 might play a certain role in the immune microenvironment of HCC (**Figures 5A**–**C**).

### Cancer pathway activity and drug sensitivity

To explore the prospects of PDSS1 in tumor drug treatment, we predicted PDSS1-related cancer pathways and the drug sensitivity of PDSS1.The results indicated that PDSS1 is probably involved in the activation of the cell cycle, DNA damage response, apoptosis, and hormone AR pathways (**Figure 5D**). Based on the GDSC and CTRP databases, low expression of PDSS1 was associated with, respectively, resistance to 25 and 109 drugs (**Figures 5E**, **F**). These results provide novel ideas for the drug therapy of HCC with high levels of PDSS1.

### PDSS1 knockdown inhibits cell proliferation, migration, and invasion *in vitro* and vivo

**Figure 6:**
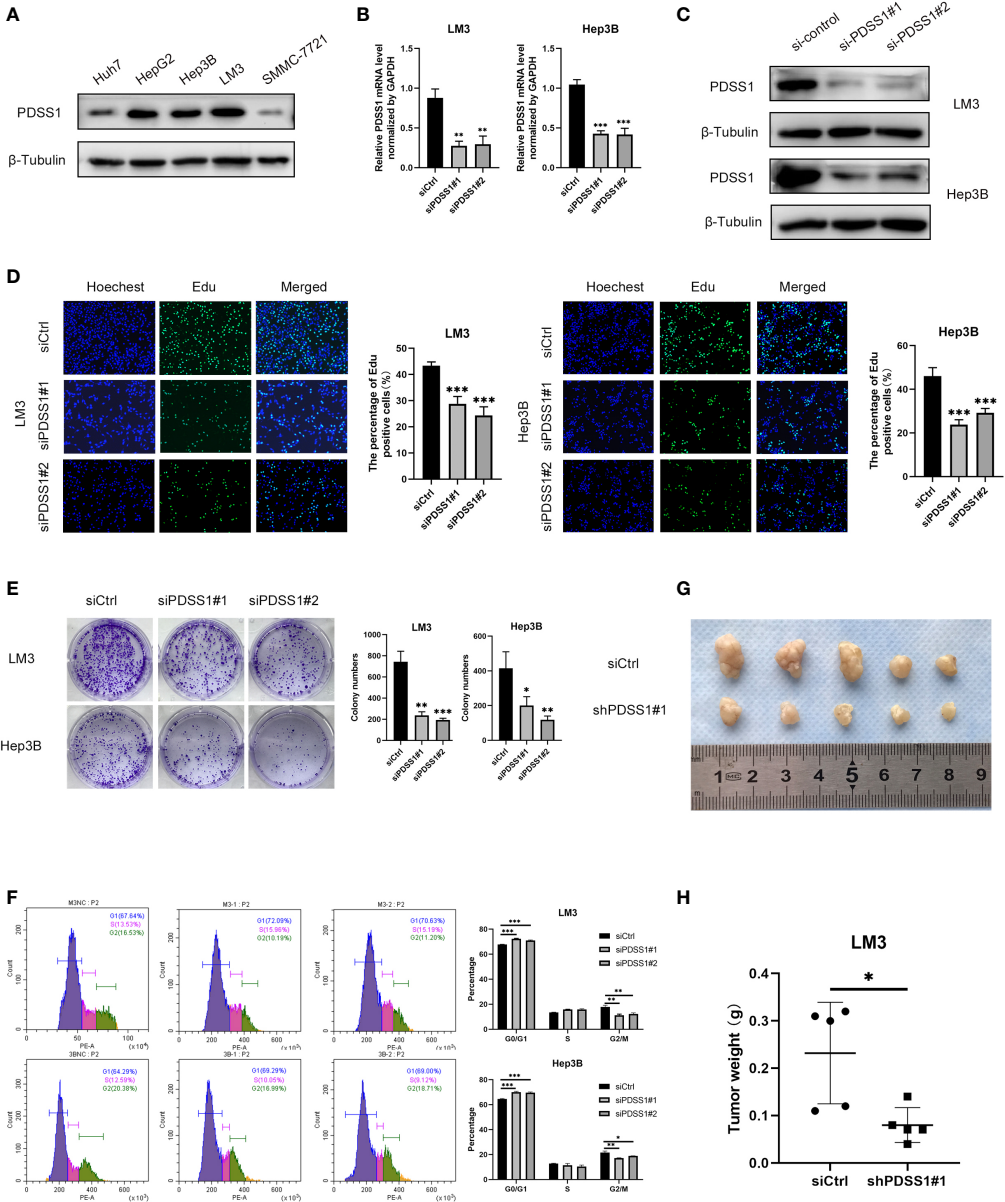
Knockdown of PDSS1 inhibited the proliferation, cell cycle, migration and invasion of HCC cells. **(A)** The level of PDSS1 was evaluated in various HCC cell lines by WB. **(B, C)** LM3 and Hep3B cells were transfected with siPDSS1, the level of PDSS1 was evaluated by qRT-PCR and WB. **(D, E)** EdU and Colony formation assays were performed to determine the proliferation of LM3 and Hep3B cells transfected with siCtrl or siPDSS1. **(F)** Cell cycle was detected in LM3 and Hep3B cells transfected with siCtrl or siPDSS1 by flow cytometry. **(G, H)** LM3 cell transfected with empty vector or shPDSS1 were injected into nude mice (n = 5) with the same concentration and amount. Data are expressed as the mean ± SD of three individual experiments. *p < 0.05, **p < 0.01, ***p < 0.001.

**Figure 7:**
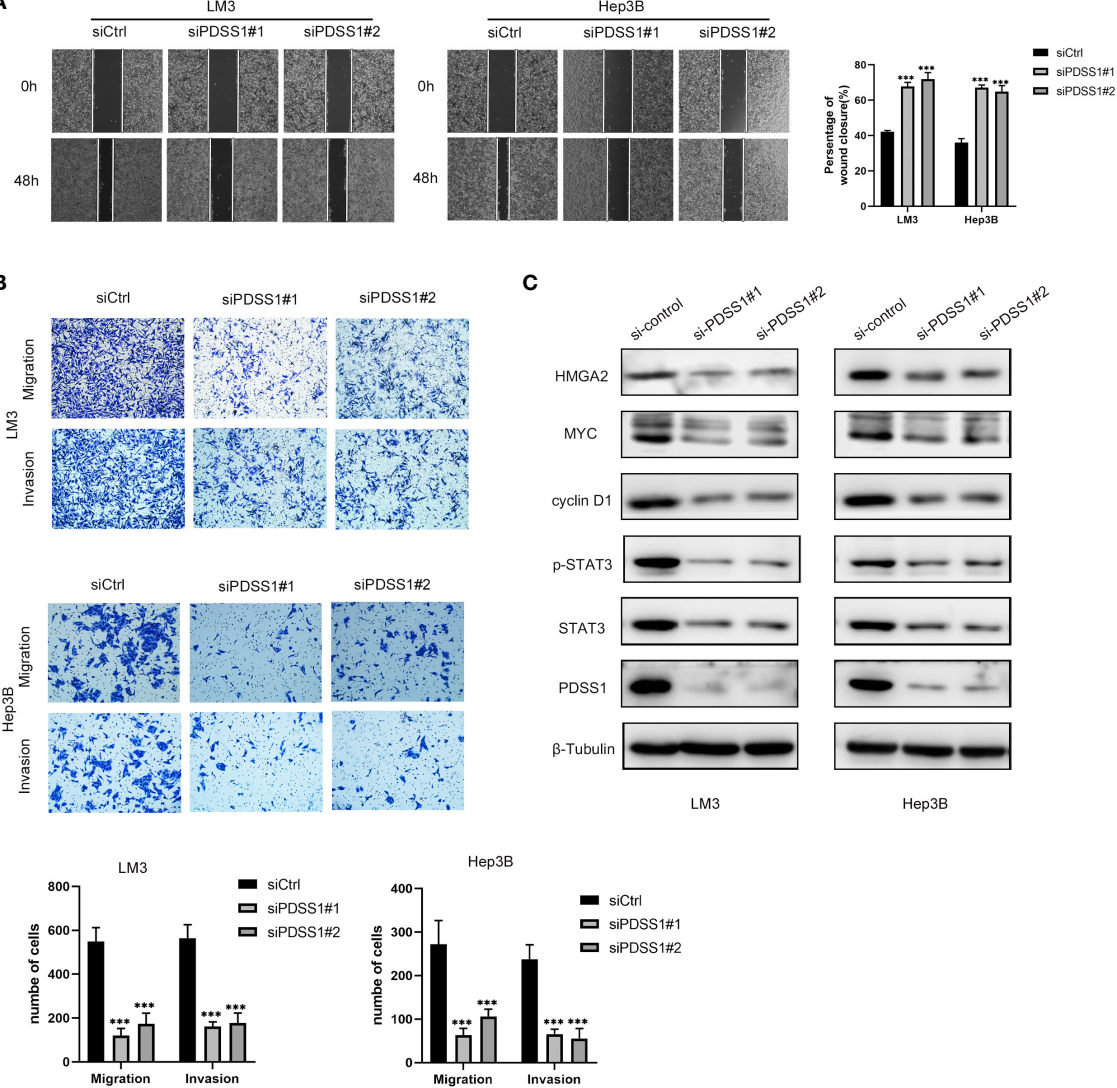
Knockdown of PDSS1 inhibited the proliferation, cell cycle, migration and invasion of HCC cells. **(A, B)** Wound healing assay and Transwell assays were used to detect the invasion and migration ability of LM3 and Hep3B cells transfected with siCtrl or siPDSS1. **(C)** The expression levels of indicated proteins were evaluated by WB in LM3 and Hep3B cells transfected with siCtrl or siPDSS1. Data are expressed as the mean ± SD of three individual experiments. ***p < 0.001.

To explore the impact of PDSS1 on HCC, we applied WB to detect the PDSS1 expression pattern in human hepatocellular carcinoma cell lines (HepG2, LM3, Huh7, Hep3B, and SMMC-7721). The results revealed that PDSS1 is expressed more highly in LM3 and Hep3B cells (**Figure 6A**). Therefore, these lines were applied for subsequent functional experiments. We silenced PDSS1 with two siRNAs. The RT-qPCR and Western blotting analyses confirmed that PDSS1 was silenced in siPDSS1#1 and siPDSS1#2 groups compared to the siCtrl group (**Figures 6B**, **C**). Next, we used EdU and colony-formation assays to test whether PDSS1 knockdown influenced the proliferation of HCC cells. The result demonstrated that silencing PDSS1 significantly suppressed the proliferation of LM3 and Hep3B cells (**Figures 6D**, **E**). Furthermore, a flow cytometry assay showed that PDSS1 knockdown induced G0/G1 phase arrest in LM3 and Hep3B cells (**Figure 6F**). To further validate the effect of PDSS1 *in vivo*, we transfected the empty vector or shPDSS1#1 plasmid into LM3 cells and injected them into the posterior flank of nude mice. The tumors that grew from the shPDSS1#1 group appeared to be smaller than those formed from the empty vector group (**Figures 6G**, **H**). In addition, the wound-healing assay indicated that PDSS1 knockdown significantly inhibited the migration of LM3 and Hep3B cells (**Figure 7A**). Transwell assays were used to explore the effect of PDSS1 knockdown on HCC cells’ migration and invasion. As shown in **Figure 7B**, the number of cells passing through the polycarbonate membrane was significantly reduced in the PDSS1 knockdown group, indicating that PDSS1 can affect the metastasis of HCC cells. The above results prove that PDSS1 may be a promoter of HCC.

### PDSS1 is related to STAT3 signaling pathway

Based on the above analysis results, we know that PDSS1 is closely related to the cell cycle. Yu et al. demonstrated that PDSS1 promotes metastasis through the STAT3 signaling pathway in triple-negative breast cancer (TNBC) (10). In order to clarify the potential mechanism of PDSS1 in HCC, we wanted to know that whether silencing PDSS1 expression can affect STAT3 level and its phosphorylation. As shown in **Figure 7C**, the levels of STAT3 and p-STAT3 were significantly decreased in PDSS1-silenced LM3 and Hep3B cells. At the same time, we tested the expression of several downstream target genes of the STAT3 signaling pathway. The results revealed that PDSS1 knockdown in LM3 and Hep3B cells led to obviously downregulated levels of cyclin D1, Myc, and HMGA2 (17–19). Taken together, these results suggest that PDSS1 may play a vital role in promoting HCC proliferation, cell cycle, migration, and invasion by activating the STAT3 signaling pathway.

## Discussion

Hepatocellular carcinoma has become a global health problem, seriously threatening human life (2, 20). After years of development, the treatment of HCC has made great progress, including hepatectomy, liver transplantation, radiofrequency ablation, TACE, molecular targeted drugs, immunotherapy, and so on (21, 22). Even so, the prognosis of HCC patients is still unsatisfactory, and the 5-year survival rate is less than 10% (23). Thus, it is essential to develop the new biomarkers and treatment strategies for HCC. Studies have predicted that PDSS1 is associated with hypoxia and immune microenvironment in hepatocellular carcinoma, but the molecular mechanism by which it acts has not been reported (24, 25). In this research, we applied a variety of bioinformatics methods and functional experiments to analyze the expression and biological functions of PDSS1 in HCC.

First, we investigated PDSS1 expression in HCC using the HCCDB and Omcomine databases. Overexpression of PDSS1 was observed in HCC tissues. We were very interested in the role of this differential expression in the occurrence and development of HCC. Subsequently, GEPIA database and Kaplan–Meier plotter analysis indicated that high PDSS1 is significantly correlated to poor prognosis (OS, DSS, and PFI). In addition, the univariate and multivariate Cox regression models told us that upregulated PDSS1 was the independent prognostic factor for poor prognosis. This provides ideas for developing clinical prognostic indicators for HCC. Next, we analyzed the co-expressed genes of PDSS1 revealed by the LinkedOmics database, after which GO functional and KEGG pathway analysis indicated that PDSS1 might be involved in the cell cycle, cell cycle checkpoints, mitotic sister chromatid segregation, p53 signaling pathway, and DNA replication. The above results point out the direction for the subsequent exploration of the oncogenic mechanism of PDSS1. In brief, the above results indicate that PDSS1 is a novel biomarker and therapeutic target for HCC.

The liver contains a large number of different types of immune cells and it is an important immune-exempt organ in the body (26). The liver can achieve immune tolerance to antigens by regulating the activation of initial T cells and a variety of immune suppression strategies. Hepatocellular carcinoma is an inflammation-related tumor, and its immune response is regulated by a variety of activation and inhibitory signal pathways. Strong immunogenicity, the diversity of the immune microenvironment, and concentrated immune cell infiltration constitute the unique immunological characteristics of the liver (27, 28). Immune infiltration analysis revealed that PDSS1 is significantly associated with a variety of immune cells in HCC. Tumor-infiltrating immune cells (TIICs) are closely related to the occurrence and development of tumors (29, 30). In our study, we found that PDSS1 in HCC was negatively related to dendritic cells (DCs, pDCs), cytotoxic cells, and CD8 T cells. DCs are a type of antigen-presenting cell which have an important regulatory effect on tumor immune response, and can positively regulate the immune response to HCC (31, 32). In addition, CD8^+^ T cells can secrete cytotoxic particles, making them a type of cytotoxic cell that plays an important role in antitumor immunity (33, 34). On the other hand, PDSS1 is significantly positively correlated with Th2 cells, TFH, and T helper cells, and they play an important role in regulating tumor immunity (35–37). In summary, the above results indicates that PDSS1 plays an important role in regulating immune cell infiltration in HCC.

When PDSS1 expression was downregulated, the ability of proliferation, migration, and invasion in HCC cell lines also decreased, and the cell cycle was blocked in the G0/G1 phase. In addition, previous literature has shown that PDSS1 promotes breast cancer progression through the STAT3 signaling pathway (10). A similar result, namely that the levels of STAT3 and p-STAT3 were significantly decreased in PDSS1-silenced cells, occurred in HCC. At the same time, the expression of downstream target genes (cyclin D1, Myc, and HMGA2) of STAT3 also decreased. The results show that PDSS1 promotes the progression of HCC through the STAT3 signaling pathway, but the specific regulatory mechanism needs to be further studied. Unfortunately, more time is needed here to explore the specific mechanisms affecting the STAT3 pathway.

## Conclusion

Our study provides multiple lines of evidence to confirm that PDSS1 is a therapeutic target and prognostic predictor of HCC and promotes the progression of HCC by regulating the STAT3 signaling pathway and immune cell infiltration. However, more experiments are needed to reveal its specific mechanisms.

## Data availability statement

The original contributions presented in the study are included in the article/**Supplementary Material**. Further inquiries can be directed to the corresponding authors.

## Ethics statement

The animal study was reviewed and approved by the Animal Ethics and Welfare Committee (AEWC) of the First Affiliated Hospital of Harbin Medical University.

## Author contributions

DX, XM, ZR, and HL conceived and designed the study. ZR and HL conducted the experiments. WY, QW, and BM conducted the data collection and analysis. ZR drafted the manuscript. All authors contributed to the article and approved the submitted version.

## Funding

This study was financially supported by the National Natural Science Foundation of China (No.82170654).

## Conflict of interest

The authors declare that the research was conducted in the absence of any commercial or financial relationships that could be construed as a potential conflict of interest.

## Publisher’s note

All claims expressed in this article are solely those of the authors and do not necessarily represent those of their affiliated organizations, or those of the publisher, the editors and the reviewers. Any product that may be evaluated in this article, or claim that may be made by its manufacturer, is not guaranteed or endorsed by the publisher.
